# Anaphylatoxin C3a receptors in asthma

**DOI:** 10.1186/1465-9921-6-19

**Published:** 2005-02-21

**Authors:** Hydar Ali, Reynold A Panettieri

**Affiliations:** 1 Department of Pathology, School of Dental Medicine, University of Pennsylvania, 240 South 40^th ^Street, Philadelphia, PA, 19104, USA; 2 Pulmonary Allergy and Critical Care Division, Department of Medicine, University of Pennsylvania, BRBII/III, 421 Curie Boulevard, Philadelphia PA 19104, USA

## Abstract

The complement system forms the central core of innate immunity but also mediates a variety of inflammatory responses. Anaphylatoxin C3a, which is generated as a byproduct of complement activation, has long been known to activate mast cells, basophils and eosinophils and to cause smooth muscle contraction. However, the role of C3a in the pathogenesis of allergic asthma remains unclear. In this review, we examine the role of C3a in promoting asthma. Following allergen challenge, C3a is generated in the lung of subjects with asthma but not healthy subjects. Furthermore, deficiency in C3a generation or in G protein coupled receptor for C3a abrogates allergen-induced  responses in murine models of pulmonary inflammation and airway hyperresponsiveness. In addition, inhibition of complement activation or administration of small molecule inhibitors of C3a receptor after sensitization but before allergen challenge inhibits airway responses. At a cellular level, C3a stimulates robust mast cell degranulation that is greatly enhanced following cell-cell contact with airway smooth muscle (ASM) cells. Therefore, C3a likely plays an important role in asthma primarily by regulating mast cell-ASM cell interaction.

## Role of complement system in the development of asthma

Asthma, a complex airway inflammatory disease, is characterized by bronchoconstriction, airway hyperresponsiveness (AHR) and remodeling. Current consensus suggests that T_H_2 cytokine producing T cells, mast cells and ASM cells play central roles in the pathogenesis of asthma [1-7]. The complement system forms an important part of innate immunity against bacteria and other pathogens. As a system of 'pattern recognition molecules', foreign surface antigens and immune complexes initiate a proteolytic pathway leading to the formation of a lytic membrane attack complex. The anaphylatoxins C3a and C5a are released as byproducts of complement activation and modulate innate immunity. Accordingly, C5a is involved in a number of inflammatory diseases such as immune-complex-mediated lung injury and sepsis [8,9]. A role for C3a in innate or adaptive immunity, however, has only been recently recognized [10].

C3a levels are elevated in bronchoalveolar lavage (BAL) fluid after segmental allergen challenge in asthmatic but not healthy subjects [11-14]. Furthermore, plasma C3a is also elevated in acute exacerbations of asthma [11]. Additionally, single nucleotide polymorphisms in C3 and C3a receptor genes increases susceptibility to asthma [15]. Collectively, these findings suggest that C3a and the cognate G protein coupled receptor (C3aR) may play a role in the development of airway hyperresponsiveness (AHR) and inflammation.

## C3a receptors in models of Airway Hyperresponsiveness

Studies with animal models provided compelling evidence for C3aR activation in the development of AHR and inflammation. Humbles et al., [12], showed that C3aR (-/-) mice in BALB/c strain are protected from AHR in response to aerosolized ovalbumin challenge following intraperitoneal sensitization with ovalbumin [12]. However, C3aR (-/-) mice developed normal airway inflammatory response with no difference in T_H_2 cytokine production and eosinophil recruitment in BAL when compared to wild-type mice. Additionally, guinea pigs with a natural defect in C3aR expression were also protected from AHR in response ovalbumin to challenge with no effect on airway inflammation [16]. These initial findings suggested that C3a modulates AHR, perhaps, via a direct action on airway smooth muscle cells [12,17,18].

Recent studies using C3aR (-/-) mice provided new insights on the role of C3a on both AHR and airway inflammation [19]. When sensitized intraperitoneally with extracts of *Aspergillus fumigatous *and challenged intranasally with allergen, these mice experienced substantial decreases in both AHR and airway eosinophilia relative to wild-type mice. Furthermore, BAL levels of T_H_2 cytokines (IL-4, IL-5, IL-13), IgE titres and mucous production were all significantly reduced in C3aR (-/-) mice. Allergen-challenged C3 (-/-) mice also display diminished AHR, lung eosinophilia and T_H_2 cytokine production when compared to wild-type mice [20]. These findings support a role of C3a receptors in the development of AHR and inflammation. However, the effect of C3aR on different phases of AHR models may depend on the nature of the allergen, method of sensitization and the strain of mice used.

## C3a generation in asthma

**Figure 1:**
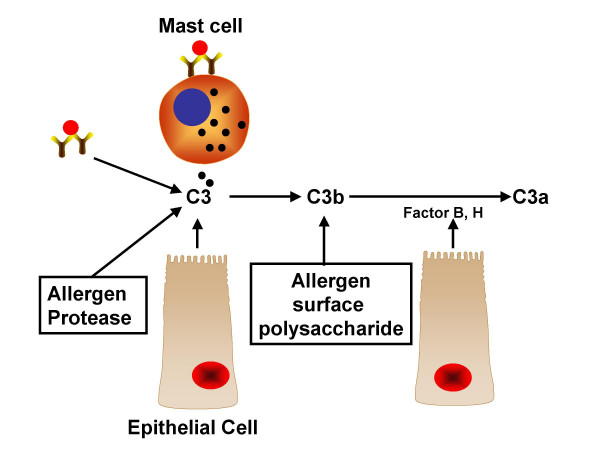
**Model for C3a generation in individuals with asthma**. C3 may be secreted from pulmonary resident cells (e.g. epithelial cells and macrophages) or derived from plasma leakage. Antibody (IgG) present in the serum of sensitized individual can form a complex with allergen to activate complement via the classical pathway. Proteases derived from allergen or released from activated mast cells are able to cleave C3 to generate C3a. Activation of alternative or the lectin pathway on the allergen together with factors B, H, and I and properdin released resident cells may generate C3a.

Increased level of C3a in BAL of subjects with asthma implies a potential role for this apaphylatoxin in promoting airway inflammation. However, the cells responsible for C3a generation and the airway effector cells stimulated by C3a remain unknown. Plausibly, antibody generated during antigen sensitization may interact with allergen to activate the classical complement pathway. Additionally, airway epithelial cells and pulmonary macrophages secrete both C3 and several components of the alternate pathway of complement (factors B, H, and I and properdin) [21-23]. Thus, activation of alternative or the lectin pathway on the allergen may also lead to the generation of C3a. It is noteworthy that house dust mite protease, allergenic extracts of *Aspergillus fumigatous *and mast cell tryptase also activate the complement pathway directly [13,24-26]. Thus, combination of different pathways likely generates C3a in the airway of individuals with asthma (Figure 1).

## C3a has little effect on allergen sensitization in models of AHR

Both antigen-presenting cells (APCs) and activated T cells express C3aR [27-30], raising the possibility C3a may regulate sensitization phase of allergic asthma. Kawamoto et al [31], recently used wild-type and C3aR-/- mice to characterize the immune response to C3a. Convincingly, C3aR deficiency had little effect on T_H_2 cytokine response to intraperitoneal ovalbumin sensitization. Furthermore, C3a had no effect on T_H_2 cytokine production in response to T cell receptor ligation. Further, Taube et al., [32] showed that administration of complement inhibitor in mice after sensitization but before allergen challenge prevented the development of AHR and blocked T_H_2 cytokine production and lung inflammation. Additionally, a small molecule antagonist of C3a receptor, when administered after sensitization but before challenge also caused significant inhibition of airway inflammation [33]. These findings suggest that the effect of C3a on the development of allergic AHR may not involve modulation of the sensitization phase of the disease.

## Relationship between C3aR and FcεRI in mast cell activation in asthma

**Figure 2:**
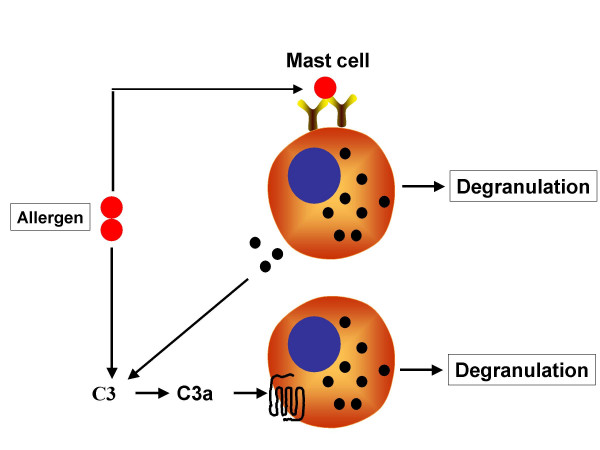
**Proposed interaction between FcεRI and C3aR leading to mast cell activation**. Allergen cross-links FcεRI on mast cells to induce degranulation. Allergen can also activate complement pathway (see Fig. 1) to generate C3a, which in turn activates its cognate G protein coupled receptors on mast cells to induce degranulation. Mast cell proteases also activates complement cascade to generate C3a. This C3a may serve to amplify mast cell mediator release.

Mast cells appear to play a pivotal role in the development of AHR and inflammation [34]. The ability of allergen to cross-link high affinity IgE receptors (FcεRI) on mast cells to induce degranulation and leukotriene generation is well documented [35,36]. Surprisingly, the role of C3a in mast cell activation remains controversial and appears to depend on the mast cells subtype. For example, murine bone marrow-derived mast cells and a rat basophilic leukemia, RBL-2H3 cells, which have been used extensively as mast cell models, do not express C3a receptors [37]. In contrast, C3a receptors are expressed in human CD34^+^-derived primary mast cell cultures [38,39], human mast cell lines HMC-1 [40,41] and LAD 2 [39] as well as murine pulmonary mast cells (Thangam, B and Ali, H, unpublished data). Interestingly, C3a is one of the most potent mast cell chemoattractants known [42,43]. C3a also induces robust mast cell degranulation [38,39] and leukotriene C_4 _generation (Thangam, B and Ali, H, unpublished data). These findings suggest that allergen induces mast cell degranulation by at least two mechanisms: cross-linking of FcεRI and via C3a generation following complement activation by allergen protease (Figure 2). Mast cell proteases also activate the complement pathway to generate C3a [26]. Therefore, C3a generation following FcεRI aggregation may amplify mast cell mediator release (Figure 2).

## Mast cell-ASM interaction in asthma

Recent studies with immunohistological analysis of bronchial biopsy specimens from subjects with asthma and those from patients with eosinophilic bronchitis provided important insight on the role of mast cell-ASM cell interaction in the development of AHR in asthma [4,44,45]. Asthma and eosinophilic bronchitis are characterized by similar inflammatory infiltrates in the submucosa of the lower airway. However, ASM infiltration by mast cells is a feature of asthma and not eosinophilic bronchitis. This difference in mast cell recruitment in asthma is associated with AHR, which is absent in in eosiniphilic bronchitis [6]. Furthermore, degranulated mast cells are detected in greater number in ASM bundles of patients who died from asthma when compared to non-asthmatic control [46]. Based on these findings, new hypothesis suggests that increased mast cell recruitment and interaction with ASM may promote release of mast cell-derived mediators that modulate resident airway cell function is asthma [4,5,44].

ASM is not only a contractile tissue that responds to mast cell-derived mediators in asthma, but also modulates mast cell function and airway inflammation. ASM cells express stem cell factor (SCF), which induce mast cell chemotaxis, survival and differentiation [47,48]. Interleukin-1β, tumor necrosis factor (TNF) and T_H_2 cytokines IL-4 and IL-13 derived cytokines also stimulate ASM to express a large number of chemokines and cytokines [49-52]. Thus, activated ASM cells secrete chemokines and cytokines that may recruit and retain mast cells into the ASM.

## C3a receptors and mast cell-ASM cell interaction

C3a has long been recognized as an agent that evokes force generation in smooth muscle. In guinea pigs, C3a-induced contraction of lung parenchyma may involve indirect effects of histamine and arachidonic acid metabolites [53]. In mice, C3a does not cause shortening of isolated tracheal strips [10]. Furthermore, C3a fails to induce AHR after intratracheal instillation in naïve mice [10]. In contrast, in mice immunized with house dust mite, subsequent intratracheal administration of C3a stimulates both AHR and airway inflammation [10]. These findings suggest that C3a-induced AHR and bronchoconstriction requires enhanced infiltration and activation of inflammatory cells, likely mast cells.

Recently, investigations showed that human mast cells but not human or murine ASM express C3aR [54]. Interestingly, incubation of mast cells with human ASM cells, but not its culture supernatant, significantly enhanced C3a-induced mast cell degranulation. Although stem cell factor (SCF) and its receptor c-kit are constitutively expressed on ASM cells and mast cells respectively, neutralizing antibodies to SCF and c-kit failed to inhibit ASM cell-mediated enhancement of mast cell degranulation. Dexamethasone-treated ASM cells however normally express cell surface SCF but were significantly less effective in enhancing C3a-induced mast cell degranulation when compared to untreated cells. Collectively, these findings suggest that cell-cell interaction between ASM cells and mast cells, via a SCF-c-kit independent but dexamethasone-sensitive mechanism, enhances C3a-induced mast cell degranulation, which likely regulates ASM function and may contribute to the pathogenesis of asthma.

**Figure 3:**
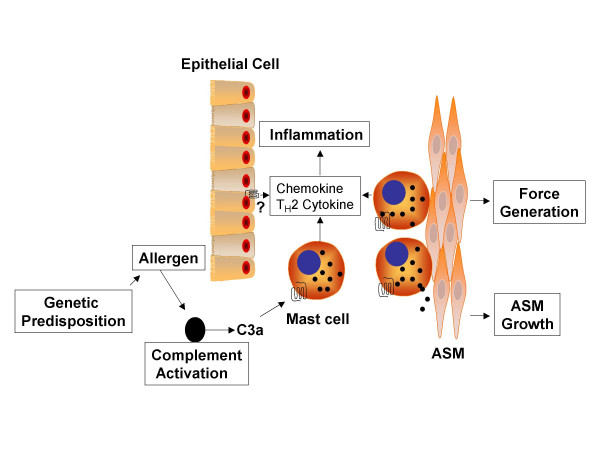
**Model for the role C3a in AHR and airway inflammation in asthma**. C3a generated in individuals with asthma (see Fig. 1) induces mast degranulation (Fig. 2) to promote ASM force generation. Chemokines and cytokines expressed by ASM recruit and retain mast cells into the ASM layer resulting in further smooth muscle dysfunction. T_H_2 cytokines and chemokines generated from mast cells (and possibly eosinophils and bronchial epithelial cells) regulate AHR and airway inflammation.

While mast cells and ASM cell interaction plays a role in AHR, airway inflammation in asthma is strongly linked to T_H_2 lymphocyte and their cytokines IL-4, IL-5 and IL-13. These cytokines play key roles in the recruitment and activation of eosinophil, mucous production and IgE synthesis. Allergen challenge of sensitized C3 (-/-) and C3aR (-/-) mice decreased production of T_H_2 cytokines in BAL and substantially reduced recruitment of T cells, eosinophils and neutrophils in lung tissue [19,20]. Furthermore, inhibition of complement activation or administration of C3aR antagonist during the effector phase of asthma substantially inhibited airway inflammation [32,33]. These findings suggest activation of C3aR is required for T_H_2 effector function in murine model of allergen-induced inflammation. Accordingly, in human mast cells, C3a stimulates the production of MCP-1, RANTES [39], IL-8 and IL-13 (Thangam, B and Ali, H, unpublished data)-cytokines and chemokines are responsible for the recruitment of T lymphocytes, eosinophils and neutrophils into the airway. Further, C3aR are expressed on basophils, eosinophils and bronchial epithelial cells [18,54-57]. Thus, interaction of a number of inflammatory and resident cells likely regulate C3a-dependent T_H_2 cytokine and chemokine production in asthma (Figure 3).

## Conclusion

Accumulating evidence suggests that C3a may play an important role in the pathogenesis of asthma. In murine models of allergic AHR and inflammation, inhibition of complement activation or small molecule antagonists of C3a receptor after sensitization but before allergen challenge inhibits airway responses. Furthermore, cell-cell interaction between ASM cells and mast cells enhances C3a-induced mast cell degranulation, which likely regulates ASM function, thus contributing to the pathogenesis of asthma. Further investigations on cellular and molecular mechanisms by which C3a modules mast cell-ASM interactions may offer novel therapeutic approaches to the treatment of asthma and airway inflammation.

## List of Abbreviations used

C3aR, C3a receptor; AHR, airway hyperresponsiveness: ASM, airway smooth muscle; BAL, bronchoalveolar lavage.

## Competing interests

The author(s) declare that they have no competing interests.
